# Individual Alpha Frequency Determines the Impact of Bottom-Up Drive on Visual Processing

**DOI:** 10.1093/texcom/tgab032

**Published:** 2021-04-26

**Authors:** Stephanie Nelli, Aayushi Malpani, Max Boonjindasup, John T Serences

**Affiliations:** 1 Neurosciences Graduate Program, University of California, San Diego, CA 92093, USA; 2 Department of Experimental Psychology, University of Oxford, Oxford OX2 6GG, UK; 3 Department of Psychology, San Diego, CA 92093, USA; 4 Kavli Institute for Brain and Mind, University of California, San Diego, CA 92093, USA

**Keywords:** alpha oscillations, dynamical systems, electroencephalography, visual perception

## Abstract

Endogenous alpha oscillations propagate from higher-order to early visual cortical regions, consistent with the observed modulation of these oscillations by top-down factors. However, bottom-up manipulations also influence alpha oscillations, and little is known about how these top-down and bottom-up processes interact to impact behavior. To address this, participants performed a detection task while viewing a stimulus flickering at multiple alpha band frequencies. Bottom-up drive at a participant’s endogenous alpha frequency either impaired or enhanced perception, depending on the frequency, but not amplitude, of their endogenous alpha oscillation. Fast alpha drive impaired perceptual performance in participants with faster endogenous alpha oscillations, while participants with slower oscillations displayed enhanced performance. This interaction was reflected in slower endogenous oscillatory dynamics in participants with fast alpha oscillations and more rapid dynamics in participants with slow endogenous oscillations when receiving high-frequency bottom-up drive. This central tendency may suggest that driving visual circuits at alpha band frequencies that are away from the peak alpha frequency improves perception through dynamical interactions with the endogenous oscillation. As such, studies that causally manipulate neural oscillations via exogenous stimulation should carefully consider interacting effects of bottom-up drive and endogenous oscillations on behavior.

## Introduction

A dynamic balance between excitatory and inhibitory neural activity leads to brain rhythms such as the prominent alpha band oscillations (~7–12 Hz) known to mediate visual information processing and behavior (van Vreeswijk and Sompolinsky 1996; Azouz and Gray 2000; Salinas and Sejnowski 2001; Brunel and Wang 2003; Draguhn et al. 2004; Fries 2005; Mazzoni et al. 2008; Atallah and Scanziani 2009; Isaacson and Scanziani 2011; Lopes da Silva 2013; Akam and Kullmann 2014; Fries 2015). Previous studies have shown that both top-down cognitive demands and bottom-up visual stimulation impact measurements of alpha oscillations such as amplitude, phase, and frequency. Specifically, top-down factors such as expectation, goal-directed attention, and working memory are associated with changes in alpha oscillations (Foxe et al. 1998; Jensen et al. 2002; Sauseng et al. 2005; Klimesch et al. 2007; Rihs et al. 2007; Yamagishi et al. 2008; Kelly et al. 2009; Haegens et al. 2011; Rohenkohl and Nobre 2011; Bosman et al. 2012), consistent with findings that alpha oscillations propagate in the feedback direction from higher- to lower-order regions along the cortical hierarchy (von Stein et al. 2000; Bollimunta et al. 2008; Fries et al. 2008; Buffalo et al. 2011; van Kerkoerle et al. 2014; Markov et al. 2014; Michalareas et al. 2016). In addition, bottom-up factors such as opening one’s eyes or viewing a salient stimulus modulate alpha oscillations in regions involved in processing the incoming stimuli (Berger 1930; Pfurtscheller 2001; Rizzuto et al. 2003; Woertz et al. 2004; Lakatos et al. 2009). However, we know relatively little about how bottom-up stimulus-evoked changes in rhythmic activity interact with endogenous alpha oscillations, even though dynamic interactions between these processes likely shift alpha frequency, amplitude, and phase (Aronson et al. 1990; Wang 2010; Nelli et al. 2017).

**Figure 1:**
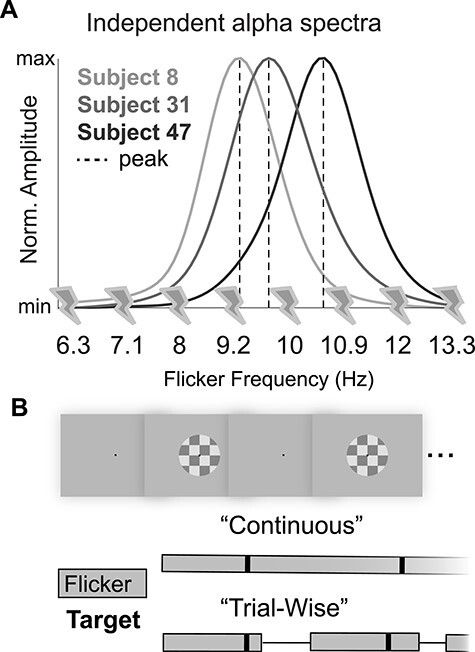
Study motivation and task design. (*A*) Three hypothetical amplitude spectra are shown and the peak alpha frequency is indicated with a dashed line. We used steady-state visual-evoked potentials at 8 alpha frequencies (SSVEPs, indicated by lightning bolts) to test whether bottom-up stimulus drive at a participant’s peak frequency negatively impacts perception (right panel). (*B*) We flickered a centrally presented checkerboard at 8 different frequencies tiling the alpha band as participants performed a contrast change detection task at fixation. Participants were either presented with a continuously flickering checkerboard during which targets were separated by a variable intertarget interval (“continuous flickering”), or a checkerboard that was removed from the screen during a short intertrial interval (“trial-wise flickering”).

To probe the interaction between endogenous alpha oscillations, we recorded scalp EEG as participants performed a change-detection task at a fixed level of difficulty intended to hold top-down factors constant (see Materials and Methods). We flickered a visual stimulus at multiple frequencies around the alpha band to drive bottom-up rhythms in visual cortex while participants performed the task (Fig. 1*A*; see Materials and Methods). Note that this manipulation is not intended to entrain the, presumably top-down, generator(s) of the endogenous alpha oscillation (Keitel et al. 2014; Haegens and Zion Golumbic 2018). Instead, this manipulation allowed us to characterize changes in behavior and endogenous oscillatory dynamics as a function of the offset between the frequency of the bottom-up stimulus drive and each participant’s endogenous peak alpha frequency.

## Materials and Methods

### Participants

Fifty-seven participants (33 in continuous version and 24 in trial-wise version, see below; 29 male) were recruited at the University of California San Diego and all data were collected at UCSD’s Perception and Cognition Lab. All participants provided written informed consent in accordance with the Institutional Review Board at UCSD. Participants were compensated $15/h for EEG. The age range of the participants was 19–30 years old, and all participants had normal or corrected to normal vision. Five participants showed poor task adherence and guessing (2 in continuous version, 3 in trial-wise version), quantified as a negative sensitivity metric for at least one of the flicker frequency conditions in the detection task. This resulted in 52 total participants for all analyses unless noted. An additional 27 participants participated (16 male, age = 21.7, range 18–32 years old) in either the continuous (768 trials, *n* = 15) or trial-wise (672 trials, *n* = 12) version of a behavioral control experiment.

### Apparatus and Stimuli

The experiment was implemented using Psychtoolbox in the MATLAB programming environment running on a Windows PC with the XP operating system. Participants were positioned 60 cm from the display and stimuli were presented on a 15-inch CRT monitor with 1024 × 768 resolution and 120 Hz refresh rate. The luminance output of the monitor was linearized in the stimulus presentation software.

### Task and Stimulus Procedure

To drive bottom-up rhythms in the alpha range, we flickered a centrally presented checkerboard at 8 frequencies encompassing the traditional alpha band (6.3, 7.1, 8, 9.2, 10, 10.9, 12, and 13.3 Hz; 25% Michaelson contrast and subtending 7.2° visual angle Fig. 1*B*). Participants were instructed to maintain fixation on a black, centrally presented fixation dot, and the target was a dimming of this fixation dot for 16 ms at an unpredictable time. We determined a contrast threshold necessary to maintain roughly 75% hit rates for each participant in a short behavioral session before EEG data acquisition. We report results collapsed over 2 versions of the task utilizing either “continuous” or “trial-wise” stimulus drive, described in detail below.

In the “continuous” version of the task, we flickered a checkerboard at one frequency for a block of time lasting 151.8 s (2.53 min), and each participant completed 2 blocks for each frequency. The order of frequencies was randomized between participants with the constraint that consecutive blocks of trials did not use the same frequency. During each block, we presented 48 targets, leading to a total of 96 target presentations per frequency. Potential target times were selected pseudorandomly from 1.2 to 148.8 s into the block with the only stipulation that consecutive targets were separated by at least 1.2 s and at most 5 s. Participants could respond at any time.

In the “trial-wise” version, frequency was chosen pseudorandomly on each trial within a block, with 48 trials per block and 10 total blocks. Fixation contrast changes only occurred on 2/3 of trials, leading to a total of 60 trials per frequency. We pseudorandomly chose target times to occur within 2208–2525 ms after the onset of the flickering stimulus to allow time for the exogenously driven rhythm to reach a stable steady state, and the flickering stimulus lasted for a total of 3000 ms. For the trial-wise version, the difference between the earliest and latest target times were fixed to be at least 95% of the total possible target onset time range (equal to 301 ms) to make sure target times were unpredictable. Participants could respond any time after the target or during the intertrial interval, which was chosen between 1750 and 2250 ms pseudorandomly for each trial.

### Behavioral Control Task

In the purely behavioral experimental control experiment, task parameters were the same as in the main task except that the central stimulus was flickered at a wider range of frequencies. One frequency was in the alpha band (10 Hz), while the rest were well outside the alpha band—0 Hz, or static, 1.5, 4.6, 10, 15, 20, and 24 Hz.

### Behavioral Metrics

For the continuous drive task version, a response was considered a correct detection (a “hit”) if it occurred from 84 to 1000 ms after a target. Any response made outside this temporal window was considered a false alarm. In the trial-wise version of the experiment, this minimum RT of 84 ms was also used. However, since participants could respond anytime during the ITI in the trial-wise version, RTs could exceed 1000 ms. A response made during one of the 33.33% of trials on which no target was presented (“catch-trials”) was considered a false alarm in the trial-wise version. From these hit rates and false alarm counts, we computed estimates of sensitivity (*d*'): *Z*(hit rate)—*Z*(FA); and bias (criterion): −0.5^*^(*Z*(hit rate) + *Z*(FA)).

### E‌EG Recording and Preprocessing

All EEG recordings took place in a sound-attenuated and electromagnetically shielded room (ETS Lindgren, Cedar Park, TX). EEG and EOG were recorded with a Biosemi Active2 System (Amsterdam, the Netherlands) using a headcap with the standard Biosemi 64 electrode layout. In addition to the 64 scalp electrodes, 1 reference electrode was placed on each mastoid (2 total), and 6 electrodes were placed around the eyes to identify and reject trials with blink and saccade artifacts. All EEG data were recorded at a sampling rate of 1024 Hz. Precise target presentation and participant response times were recorded as triggers in the EEG data file.

After data collection, data from the scalp electrodes were re-referenced to the algebraic mean of the 2 mastoid electrodes and raw time series from each electrode were bandpass filtered between 0.25 and 55 Hz to attenuate drift and 60 Hz line noise. Trials were excluded if the subject displayed an artifact or eyeblink 200 ms before or after a target (defined as either a difference in more than 90 millivolts between sensors placed above and below the eye or an amplitude greater than 95% of timepoints). Data were either aligned to the nearest “on” frame of the flickering stimulus (for steady-state visual-evoked potential, or SSVEP, analyses) or to the target (event related potential, or ERP, analyses) before epoching. This was done because measuring neural activity evoked by the steady-state flickering stimulus relies on precisely estimating the phase-locked response, while ERP analyses depend only on the time of target onset.

### Peak Endogenous Frequency Estimation

In addition to the main task, we recorded scalp EEG data while participants rested in order to independently estimate each participant’s endogenous peak alpha frequency. Participants first completed half of the experimental blocks, which took approximately 20 min for both experiments (8 blocks for the continuous flicker, 5 blocks for the trial-wise flicker). Then, participants were instructed to relax and fixate on a central fixation point for 3 min and then subsequently asked to close their eyes and relax for 3 min. We report peak frequency estimates from this latter, 3-min eyes-closed portion of the data due to both precedent in the literature and higher signal-to-noise ratios (SNR, see below, and refs. (Zauner et al. 2012; Cohen 2014; Samaha and Postle 2015). We computed spectra from raw, unfiltered data, which were epoched into 2000 randomly chosen artifact free 4 s intervals. Each epoch was chosen by pseudorandomly selecting a start time across the entire 3 min of recording, so epochs could be partially overlapping. Timepoints for which a channel was $\pm 3$ standard deviations from the channel’s average were flagged for exclusion due to artifacts. If the mean $\pm 3$ standard deviation was below 50 uV or exceeded 100 uV, we instead used 50 uV or 100 uV, respectively, to flag artifact timepoints (Itthipuripat et al. 2015). An entire channel was considered “bad” if more than 1.5% of total timepoints exceeded this cutoff and was not used to select epochs; otherwise, epochs were excluded from selection if they included artifact-flagged timepoints in any channel (average channel/epoch rejection counts were 9.7/28.2 and 9.4/20.4 averaged over participants in the continuous and trial-wise versions of the experiment, respectively) (Itthipuripat et al. 2015). We extracted complex coefficients for these 2000 epochs at frequencies from 2 to 20 Hz in steps of 0.1 Hz using overcomplete wavelet Morlet decomposition with 0.15 fractional bandwidth. We used wavelet decomposition as opposed to a windowed FFT so that we could control the filter characteristics and spacing in the frequency domain (note that fractional bandwidth is equivalent to the bandwidth at full-width-half-max divided by the center frequency) (Herrmann et al. 2005; Jahankhani et al. 2006). We then estimated endogenous amplitude at each frequency as the absolute value of the complex coefficients for each epoch before averaging over all timepoints and epochs (Welch 1967).

Peak alpha frequency was estimated from these spectra by extracting the frequency with maximal amplitude within the range of 7–12.5 Hz. As the max function will take the maximum value even if the signal is purely noise, for each participant, we discarded channels that had low SNR, or low *relative* alpha amplitude, at the estimated maximal frequency. At each channel, we assessed SNR at the estimated peak frequency by identifying inflection points in the amplitude spectrum by looking for a sign change in it's derrivative (i.e. where the alpha bump begins and ends). If these inflection points were ill-defined and there was more than one definitive sign change in the derivative, we used ±3 Hz from the frequency with maximal amplitude (e.g., if the estimated peak frequency was 11.5 Hz, then we also estimated amplitude at 8.5 and 14.5 Hz). We then computed SNR as the percent increase of alpha amplitude at peak compared with these points and defined a cutoff of SNR = 1 to identify channels where there is no apparent alpha “bump” that rises above amplitude at neighboring frequency bands. Thus, channels with an SNR < 1 were considered to have no identifiable peak alpha frequency. Note that this method will flag channels with multiple alpha peaks as well as channels in which alpha amplitude does not rise above the baseline spectrum. To compute stable and reliable peak alpha frequency estimates, we averaged endogenous frequencies across Oz and 4 neighboring electrodes in the main text, ignoring “NaN” values (POz, Iz, O1, and O2; consistent with previous literature) (Cohen and Gulbinaite 2017).

### Rhythmic Entrainment Source Separation (RESS)

**Figure 2:**
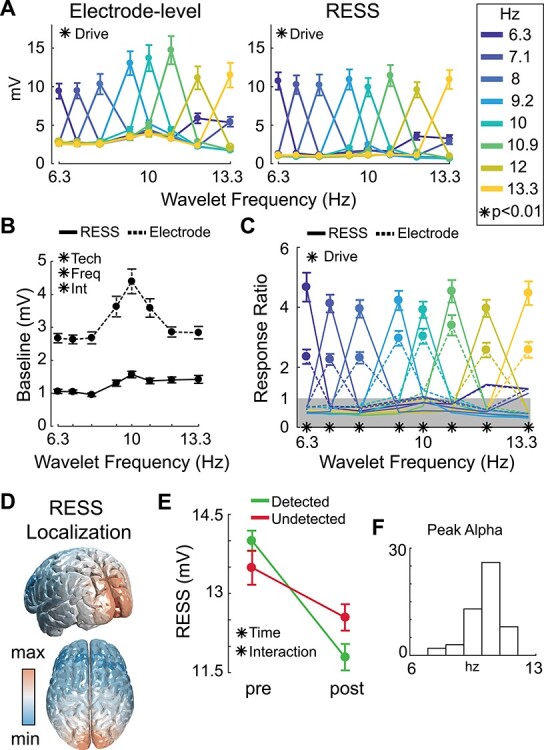
Exogenously driven alpha band rhythms. All errorbars indicate between-participant SEM. (*A*) Average amplitudes within each flicker frequency are plotted for electrode-level (left) and RESS (right) pipelines. Line shading indicates flicker frequency, ^*^ indicates significant main effect of exogenous drive at *P* < 0.01 using one-way repeated measures ANOVA. Right legend indicates the flicker frequency. (*B*) Amplitude at frequencies that were not presented were compared between electrode-level (dotted black) and RESS data (black). ^*^ indicates significant main effect of analysis technique and an interaction between technique and frequency using a 2-way repeated measures ANOVA with technique and frequency as factors. (*C*) Response ratios computed as the amplitude at flickered frequencies divided by amplitudes at frequencies that were not presented. Dotted lines used for electrode level, solid lines used for RESS. ^*^ indicates significant main effect of exogenous drive at each frequency using paired t-tests at *P* < 0.01. Gray line at one indicates that amplitude at a particular frequency was equal to the average of all other frequencies. (*D*): Projection of topographic weights used to isolate the driven rhythm using RESS (Cohen and Gulbinaite 2017). Black areas indicate high activation averaged over all subjects and flicker frequencies. (*E*) The amplitude of driven rhythms decreases posttarget, an effect that was greater on trials in which the target was correctly detected. ^*^ indicates significance at *P* < 0.01 using 2-way repeated measures ANOVA with time interval and detection as factors. Amplitude averaged over all stimulation frequencies at −1000:−500 ms and +500:+1000 ms time windows to avoid target evoked effects. (*F*) Histogram of peak alpha frequencies for each subject as determined from resting portion of the task (see Materials and Methods).

We used a source separation technique optimized for SSVEP in order to both evaluate the strength of bottom-up sensory drive and to investigate the dynamics of the endogenous, ongoing alpha rhythm without confounding it with the bottom-up sensory drive. We briefly describe the rhythmic entrainment source separation (RESS) procedure we used to obtain spatial filters and amplitude estimates, but note that details and MATLAB scripts are provided in (Cohen and Gulbinaite 2017). For each flicker frequency, covariance matrices of single trial data were computed on data filtered at that frequency (covariance at, or CA) as well as ±1 Hz from the flicker frequency (covariance surround, or CS). We use this relatively narrow range since we wanted to isolate the SSVEP frequency of interest, which is experimentally manipulated and thus known a priori. We then found the eigenvalues (*e*_vals_) and eigenvectors (*e*_vecs_) such that CA^*^e_vecs_ = CS^*^e_vecs_^*^e_vals_ using MATLAB’s built-in “eig” function. For each participant and frequency, we extracted the eigenvector with the maximum eigenvalue as a scalp map or spatial filter for that SSVEP frequency. These spatial filters over all electrodes, defined as the maximal eigenvector, were used in several analyses and figures, as described below. First, we obtained single-trial RESS timecourses by filtering data at each timepoint and trial by that scalp map (e_vec_^*^data). Next, PCA was computed on data with the RESS scalp map projected out (described in PC Subspace, Trajectories, and StatisticsMaterials and Methods section). Finally, projections onto the brain seen on the brain maps in Fig. 2*D* were generated by correlating the spatial filter (i.e., maximal eigenvector) with a leadfield matrix developed for the Biosemi 64 electrode montage by (Cohen and Gulbinaite 2017). A leadfield matrix is an approximation of how sensitive each lead (electrode) likely is to underlying dipole sources, and the leadfield itself and more information about its calculation are found in (Cohen and Gulbinaite 2017). We assessed whether the extent of this spatial filter differed with driving frequency by quantifying the number of electrodes with loadings greater than 75, 80, 85, 90, and 95th percentiles. We found no main effect of flicker frequency *F*(7, 357)s = 1.7,1.2,1, 0.5, 1.3 with *P*’s = 0.09, 0.31, 0.42, 0.78, 0.26, respectively, with *P* values determined by comparing to a null distribution of *F* values obtained by running ANOVAs on counts after randomizing flicker frequencies 10 000 times.

### Time Frequency Analyses

To determine whether we selectively drove bottom-up rhythms at the flicker frequencies, both RESS and raw data were subjected to wavelet decomposition. Data were averaged over all trials of each flicker frequency and then complex coefficients were extracted using overcomplete Morlet wavelet decomposition with fractional bandwidth of 0.1 at each of the flicker frequencies. Note that we used a fractional bandwidth of 0.1 due to the very narrow spacing between flicker frequencies. For statistics comparing drive between detected and undetected trials, we randomly resampled the minimum number of trials 100 times to balance between detected and undetected trial counts. Five participants had ≤1 trial in which the target went undetected for at least one of the flicker frequencies and thus were excluded from these specific statistical comparisons.

### Behavioral Peak-Centering

In order to directly assess modulations in behavior with respect to each participant’s particular alpha frequency, we centered behavioral metrics for each participant on their peak alpha frequency as estimated during a separate eyes closed resting period. To do this, we used a 1D shape-preserving piecewise cubic interpolation (“pchip”) because this algorithm interpolates locally, meaning it is not subject to overshoots or introducing oscillations (in the case that the data are not smooth) (Fritsch and Carlson 1980). Specifically, the pchip interpolating function *p*(*X*(*j*)) = *Y*(*j*), satisfies the following conditions:

1. *p*’ is continuous.
2. *p*’(*X*(*j*)) is chosen so that *p*(*x*) respects monotonicity, meaning if the data are monotonic so is *p*(*x*).

We chose to interpolate from −1.5 to +1.5 Hz around each participant’s endogenous peak frequency in steps of 0.5 Hz for all peak-centering analyses. This allowed us to peak-align all 52 participants under the criterion that that there is at least one data point (i.e., stimulation frequency) less than and greater than each of our participant’s peak alpha frequency. Furthermore, this choice of a relatively narrow frequency range was motivated by the scale of spontaneous shifts in endogenous alpha frequency associated with enhanced cognitive performance (Samaha and Postle 2015; Mierau et al. 2017; Nelli et al. 2017).

To confirm that the exact choice of interpolation algorithm does not alter our conclusions, we repeated peak-centering analyses using a wider interpolation band, a spline interpolation technique, and a model that did not include a linear term (Supplementary Fig. 4, Supplementary Table 1).

### Nonlinear Regression Model

We next quantified whether there were modulations in behavior due to changes in the frequency of bottom-up stimulus drive with respect to the endogenous peak alpha frequency. To do this, we fit each behavioral metric separately for each participant using a model with one linear regressor and 2 sinusoidal regressors of one cycle each in order to capture nonlinear modulations in behavior in a relatively assumption-free manner $(y={\beta}_{\mathrm{sin}}\sin x+{\beta}_{\mathrm{cos}}\cos x+{\beta}_{\mathrm{lin}}x+\mathrm{constant})$. The cosine regressor reached a minimum at the peak frequency, while the sine regressor was simply an orthogonal cycle over the period defined by the interpolation range (i.e., phase shifted by 90°). We also included an intercept and a linear term in the model and estimated beta $(\beta )$ values separately for each behavioral metric and participant. To determine significance, we randomized the frequency axis 5000 times and estimated $\beta s$ on each of these iterations. We then performed *t*-tests against zero on both randomized and observed $\beta s$, and computed *P* values as the probability of obtaining the observed *t*-statistic compared with *t*-statistics obtained by randomized frequency axes.

Finally, our nonlinear model appeared to capture the full range of frequencies equally, as fit residuals were not impacted by whether the flicker frequency was above, at, or below peak alpha (one-way repeated measures ANOVA on average residuals below, at, and above peak alpha: *F*(2,155)‘s = 0.80, 2.19, 0.66, 1.04 with *P*’s = 0.45, 0.12, 0.52, 0.36 for hit rates, RTs, sensitivity, and bias respectively; *F*(2,128) = 0.09 with *P* = 0.91 for false alarms as this was computed excluding 9 participants with no false alarms at at least 1 flicker frequency). Finally, we replicated our main findings with both a reduced sinusoidal model and a polynomial model (Supplementary Tables 1 and 2).

### Behavioral Distributions and K-Means Clustering

**Figure 3:**
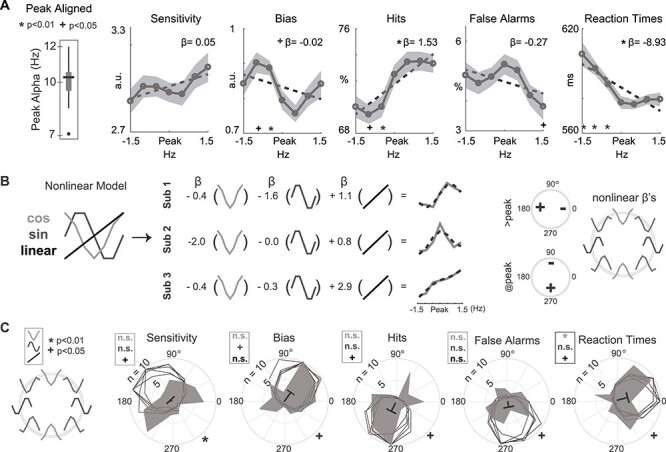
Modeling of behavioral data with a sine/cosine model. (*A*) Behavioral metrics for each participant were centered on their respective peak alpha frequency. *T*-tests were performed for behavior at each frequency in relation to the peak alpha frequency, significance is marked with ^*^ for *P* < 0.01 and + for *P* < 0.05 marked along *x*-axis. Shaded areas indicate between-subject SEM. (*B*) Each participant’s behavioral data was fit separately using 2 nonlinear regressors (sine and cosine) and a linear regressor (left panel). Hit rate fits for 3 example participants are shown, with beta values for each regressor and the resultant fit (black dotted lines, middle panels). Actual behavioral data (solid gray lines) are plotted alongside these fits. Right panel: nonlinear βs can be visualized simultaneously on a polar plot. Regions with + indicate that drive at or above peak alpha is associated with an increase in a particular behavioral metric. Regions with—indicate decreases when participants receive drive at or above their peak alpha. Example regressors are plotted along the perimeter. (*C*) Visualization of nonlinear regressors. The polar plot legend is reproduced (left panel). Radial distance indicates the number of participants for the gray shaded histograms (inner line indicates 5 participants; outer line indicates 10). Black line corresponds with the mean vector over all participants, where the outermost radial line labeled with *n* = 10 is the maximal vector length. + indicates significance at 0.05 and ^*^ indicates significance at 0.01 computed based on *t*-tests against zero on βs for the regressor indicated by shading color—light gray is cosine, gray is sine, and black is linear. Unfilled gray histograms are 5 examples of Von Mises distributions. At the bottom right of the polar plots, + indicates that at least 95% of the sampled Von Mises distributions were significantly different from the observed distribution at *P* < 0.05, ^*^ indicates that at least 99% sampled Von Mises distributions were significantly different from the observed distribution at *P* < 0.01.

When plotted on a circular histogram, beta weights from the sinusoidal regression model indicated a bimodal distribution in how bottom-up alpha drive interacts with each participant’s peak frequency to impact behavior (Fig. 3). We quantified this multimodal distribution with circular Kolmogorov–Smirnov tests. As a standardized circular equivalent to testing against a normal distribution, we compared the observed distributions to 1000 Von Mises distributions randomly sampled with equal mean and variance as our observed behavioral distribution. To investigate these groupings, we performed *K*-means clustering on the nonlinear (sine and cosine) beta values for all behavioral metrics estimated from our regression model. This 52 participant ×10 feature matrix of beta values was the input to MATLAB’s *k*-means algorithm, and we used the default squared Euclidean distance as the minimization distance metric. We iterated through the process 100 times, and within each iteration, we reinitialized the centroid cluster positions 5 times to find a lower local minimum. The participant grouping with the lowest within-cluster sum of point-to-centroid distances was chosen out of these 100 iterations.

To assess the number of clusters that were most parsimonious with the data, we repeated the above process using cluster sizes ranging from 1 to 6. Both the sum and mean of all within-cluster sum of point-to-centroid distances received the largest reduction from 1 to 2 clusters compared with any number of clusters beyond 2 (Supplementary Fig. 2). Additionally, we computed silhouette statistics for our cluster assignments to assess goodness of fit. This metric compares the relative distances between each data point to other points in its assigned cluster and between each data point to points in the next nearest cluster. Values close to one indicate that a given observation is a good fit for its cluster (Rousseeuw 1987). Specifically, for each participant *i*, silhouette value *s* is defined as:

$$s(i)=\frac{b(i)-a(i)}{\max \left\{b(i),a(i)\right\}};-1\le s\left(\mathrm{i}\right)\le 1$$

where *a*(*i*) is the average distance between i and other data within the same cluster, and *b*(*i*) is the smallest average distance of *i* to all points in any other cluster. Silhouette values for 2 clusters were highest, with sum = 20.03 and mean ± SE = 0.39 ± 0.02 (paired *t*-tests on silhouette values: *t*(51)‘s = 3.4, 3.1, 2.6, and 3.9, with *P*’s = 0.001, 0.003, 0.01, and 0.0003 for clusters from 3–6, respectively). For these reasons, we chose to separate participants into 2 clusters.

To check the robustness of our *k*-means clustering effect, we repeated this partitioning with 3 alternative modeling pipelines. First, using a spline interpolation scheme to center the behavioral data on each participant’s peak alpha frequency (instead of the *p*-chip interpolation in the main analysis), we found that 49 of the 52 participants were partitioned into the same group. Second, if we ran the same regression model without a linear term, we found that 47 of the 52 participants were partitioned into the same group. When we interpolated from −2 to +2 Hz (instead of −1.5 to 1.5 Hz) using the *p*-chip algorithm, we found that 45 of 52 participants were partitioned into the same group (Supplementary Table 1). Finally, we also replicated our main findings using sensitivity as the only input to the clustering algorithm (Supplementary Fig. 5). T-SNE plot used for visualization was computed using MATLAB’s built-in tsne algorithm.

### PC Subspace, Trajectories, and Statistics

To address whether the observed behavioral effects were associated with the impact of bottom-up stimulus drive on the dynamics of the endogenous alpha oscillation, we defined an independent “endogenous alpha” principal component (PC) state space using data from the resting block. First, the full 3 min of resting data was bandpass filtered from −1 to +1 Hz around each participant’s peak alpha frequency using a third-order zero-phase digital Butterworth filter. We removed timepoints for which EEG amplitude was ±3 standard deviations away from the mean from the data. We then correlated the resulting electrode-by-time matrix with itself and performed PCA on this electrode-by-electrode correlation matrix as in Baria et al. 2017 and retained the first 10 PCs that explained the most variance, PC_vecs_.

Then, for each participant, we computed PC trajectories on task-engaged data that had the RESS scalp map projected out. Specifically, we took the eigenvectors (*e*_vecs_) that did not account for bottom-up drive in the RESS pipeline and task engaged (*data*) onto these eigenvectors: no_ressDat = pinv(*e*_vecs_*^T^*)^*^e_vecs_^T^^*^data (pinv indicates the Moore–Penrose pseudoinverse). This step was done to isolate the nonentrained components of the task-engaged data. Note that without the removal of the first RESS component from *e*_vecs_, this equation is equal to the identity matrix. All of these methods were performed exactly as outlined in (Cohen and Gulbinaite 2017). Both this step and using resting data to form the PC subspace were done with the aim of isolating the task-engaged endogenous alpha oscillation from exogenously driven rhythms. We then filtered this eigen-projected data from −1 to +1 Hz around each participant’s peak alpha frequency using a third-order zero-phase digital Butterworth filter. We projected each timepoint and trial of this task-related, but RESS subtracted, data from all channels into the space formed by the span of the first 1–10 PCs of the independent resting data, which were defined as described in the previous paragraph. Thus, task-engaged data without RESS were projected into the PC subspace defined on the independent resting data using PC_vecs_′^*^ no_ressDat. Finally, we calculated both the Euclidean distance and velocity for each trial of the task-related data in the independent endogenous alpha state space and then averaged over either detected or undetected trials.

Because our results showed significant between-participant differences in how flicker frequency and peak alpha frequency interact to impact behavior, we predicted state space velocity using a linear mixed effects model. This model included flicker frequency, peak alpha frequency, and their interaction as fixed effects, and each participant as a random effect, which may also allow for better generalization from our current sample of 52 participants to the general population. We assessed the empirical significance of each term by comparing *t*-values computed by testing observed beta coefficients against zero to *t*-values obtained after 5000 random assignments of condition labels. Degrees of freedom for these test statistics were computed as N-P where N was the number of observations and P was the number of fixed effects, according to MATLAB’s default “residual” setting. We also ran linear mixed effects models using the same design matrix to predicted event-related potential (ERP) amplitude and phase locking index (PLI computed as in^14,67^) instead of state space velocity. Endogenous ERP and PLI data were also quantified from task-engaged data after projecting the RESS scalp map out. We then averaged ERPs and PLIs over the 5 posterior electrodes used in previous analyses (O1, Iz, Oz, POz, O2).

## Results

### Task Design and Behavior

We flickered a centrally presented checkerboard at 8 frequencies spanning the alpha band while participants performed a target detection task at fixation (6.3, 7.1, 8, 9.2, 10, 10.9, 12, and 13.3 Hz; Fig. 1*B*). The target was a 16 ms decrease in the luminance of a black, centrally presented fixation dot. Participants (*N* = 52) completed a short behavioral session to determine a luminance decrement threshold before the EEG recording session. This resulted in average hit rates of 72.27 ± 15.98% during the EEG recording session (false alarm rates = 4.69 ± 6.55% and RTs = 585.06 ± 216.14 ms, respectively; mean ± SD; see Materials and Methods). Alongside these measures, we also calculated each participant’s ability to distinguish the target from noise (sensitivity or d’), and their tendency to report a target as “present” regardless of ground truth (bias). Overall, participants were able to dissociate signal from noise with relatively conservative criteria for reporting a target (sensitivity: 2.94 ± 0.86 SD, bias: 0.8 ± 0.43 SD).

Participants completed 2 slightly different versions of the task; in one version, the stimulus was flickered at one frequency for an entire block of trials (“Continuous flickering”), and in the other version, the flicker frequency changed on every trial (“Trial-wise flickering”). We found significant response to exogenous drive and consistent behavioral effects in the 2 versions, and so report results collapsed across these versions in the main text (see Materials and Methods and Supplemental Results).

### Rhythmic Responses to Exogenous Drive

To confirm that our flickering stimulus drove brain rhythms at the intended frequency, we employed an algorithm designed to isolate the topography of exogenously driven rhythms (RESS; see Materials and Methods) (Cohen and Gulbinaite 2017). We validated these results against the traditional approach of using the raw EEG signal from an occipital electrode (Oz; see Materials and Methods). For both RESS and electrode-level data, we calculated the average amplitude at each flicker frequency in a 2000 ms window around target presentation (−1000 ms to +1000 ms). This produced 2-dimensional SSVEP frequency × calculated frequency amplitude matrices for each participant (i.e., for 6.3 Hz SSVEP trials, we calculated the amplitude at all 8 alpha frequencies; for 7.1 Hz SSVEP trials, we calculated amplitude at all 8 frequencies, etc.). Both electrode-level and RESS analyses confirmed above-chance rhythmic responses at each flicker frequency (Fig. 2*A*; ME of stimulation in one-way repeated measures ANOVA; RESS: *F*(1,103) = 75.96, *P* < 10^−10^; Electrode: *F*(1,103) = 48.67, *P* < 10^−8^).

### RESS Better Isolates Exogenously Driven Rhythms from Endogenous Oscillations

Although both electrode-based and RESS methods (see Materialsand Methods) effectively estimated SSVEP amplitudes, electrode-based amplitude estimates included more “bleed” from endogenous alpha rhythms (endogenous alpha mean ± SD = 10.08 ± 0.92; Fig. 2*A*—increase in amplitude estimates at 10 Hz: ME of wavelet frequency: *F*(7,357) = 13.6, *P* < 10^−14^, Frequency—Technique interaction: *F*(7,357) = 10.33, *P* < 10^−11^; Fig. 2*B*—baseline increase at nonpresented frequencies: ME of technique: *F*(1,51) = 216.25, *P* < 10^−15^; see Materials and Methods).

We calculated response ratios as the amplitude at each flicker frequency divided by the amplitude at all other frequencies and found that using RESS resulted in more specificity, as expected (Fig. 2*C*; ME of technique: *F*(1,357) = 237.6, *P* < 10^−15^; paired *t*-tests: higher ratios at driven frequencies 3.79 ≤ *t*(51)‘s ≤ 9.8, *P*’s ≤ 0.0003; lower ratios at nondriven frequencies −7.1 ≤ *t*(51)s ≤ −2, *P*’s ≤ 0.044). Spatial maps obtained through RESS indicated a posterior topography, consistent with our intent to drive bottom-up rhythms in the alpha band (Fig. 2*D*). This map is averaged over all flicker frequencies as we did not find a main effect of frequency on the spatial spread of the exogenously driven rhythms (one-way ANOVA ME of flicker frequency: *F*(7, 357)s ≤ 1.7, *P*’s ≥ 0.09; see Materials and Methods). Thus, it appears the bottom-up stimulus-driven rhythms are distinct and separable from the endogenous alpha oscillation, consistent with previous results finding that both top-down and bottom-up manipulations impact alpha rhythms (von Stein et al. 2000; Pfurtscheller 2001; Rizzuto et al. 2003; Woertz et al. 2004; Klimesch et al. 2007; Bollimunta et al. 2008; Fries et al. 2008; Lakatos et al. 2009; Buffalo et al. 2011; Rohenkohl and Nobre 2011; van Kerkoerle et al. 2014; Markov et al. 2014; Michalareas et al. 2016; Nelli et al. 2017; Haegens and Zion Golumbic 2018). Note that we revisit the RESS analysis later to further explore the dynamics of the interaction between endogenous and exogenously driven alpha oscillations.

Lastly, we replicated classic results indicating that SSVEP amplitudes reflect attentional engagement (Morgan et al. 1996; Müller et al. 1998) (Fig. 2*E*). Specifically, we observed increases in amplitude before a target and on trials during which the target was detected (ME of pre- vs. posttarget: *F*(1,45) = 13.3, *P* < 0.001; time epoch × detection interaction: *F*(1,45) = 15.2, *P* < 0.001). These changes in amplitude suggest that the bottom-up stimulus driven rhythms are subject to modulation by top-down attentional processes often associated with endogenous alpha oscillations. We next explore whether there is an interactive impact of the endogenous and driven rhythms on behavior.

### Behavior in Response to Bottom-Up Drive Aligned to Endogenous Alpha Frequency

To assess whether behavior is impacted by an interaction between bottom-up drive at different frequencies and endogenous alpha oscillations, we first aligned behavioral metrics to each participant’s peak alpha frequency as estimated during a separate block of EEG recording in which participants were not viewing a stimulus or performing any task (Fig. 3*A*: see Materials and Methods; nonshifted plots in Supplementary Fig. 1*A*). Bottom-up exogenous drive at peak alpha as compared with below peak alpha generally improved behavior through reducing bias, increasing hit rates, and decreasing reaction times (Fig. 3*A*; paired *t*-tests between peak and each driving frequency: bias: −1 Hz *t* = −2.38, *P* = 0.02, −0.5 Hz *t* = −4.66, *P* < 0.0001; hit rates: −1 Hz *t* = 2.48, *P* = 0.017, −0.5 Hz *t* = 2.90, *P* = 0.006; RT: −1.5 Hz *t* = −2.73, *P* = 0.009, −1 Hz *t* = −2.91, *P* = 0.005, −0.5 Hz *t* = −3.23, *P* = 0.002; false alarms: +1.5 Hz *t* = 2.31, *P* = 0.025; all other *P*’s > 0.05). Additionally, we found linear trends for enhanced performance with increasing flicker frequency (multiple linear regression betas/*P*’s: sensitivity: 0.04/0.003; bias: −0.008/0.2; hit rates: 0.93/<10^−8^; FA: −0.09/0.28; RTs: −4.5/<10^−9^). To determine whether linear trends were specific to drive in the alpha band or extended to neighboring frequency bands, we ran a behavioral version of the task using a wider range of frequencies (0 Hz, or static, 1.5, 4,6, 10, 15, 20 and 24 Hz; see Materials and Methods). We found only a slight decrease in RTs as a function of increasing frequency, indicating that the effects observed in the main study do not extend to other frequency ranges (Supplementary Fig. 3; linear regression on 7 stimulation frequencies>0; sensitivity: *P* = 0.16; bias: *P* = 0.19; Hits: *P* = 0.57; false alarms: *P* = 0.13; RT: *β* = −0.64 ms, *P* = 0.012).

Although the reported behavioral modulations appear to be specific to the alpha band, it was not clear whether the observed behavioral patterns were due to linear trends or nonlinear modulations with respect to peak alpha frequencies. Indeed, simply comparing peak and off-peak frequencies and computing linear trends poorly captured the complex behavioral patterns at the single subject level (Fig. 3*B*).

To better capture different patterns of behavior, we designed a model with sine and cosine regressors along with constant and linear terms (Fig. 3*B*; Supplementary Table 1; see Materials and Methods). The sin and cosine functions were chosen because they form a basis set that can compactly summarize nonlinear patterns regarding the impact of exogenous drive on behavior (Fig. 3*B* right panel). For example, positions at the top of these polar plots correspond with decreases in the behavioral measure with exogenous drive at peak alpha frequency. This analysis revealed that RTs were actually faster with bottom-up alpha drive at peak alpha compared with above or below peak alpha (Fig. 3*C*, Black vectors indicate mean over all participants; $\beta s$ for nonlinear regressors are in z-score units; ${\beta}_{\mathrm{cos}}=0.445\pm 0.16\ \mathrm{SE}$, *P* = 0.005). We also observed a marginal decrease in bias with drive above peak alpha (${\beta}_{\mathrm{sin}}=0.38\pm 0.17\ \mathrm{SE}$, *P* = 0.037), and marginal linear trends in hit rates, RTs, and sensitivity (${\beta}_{\mathrm{lin}}=0.41\pm 0.19\ \mathrm{SE}$, *P* = 0.033; ${\beta}_{\mathrm{lin}}=-0.64\pm 0.24\ \mathrm{SE}$, *P* = 0.012*;*${\beta}_{lin}=0.46\pm 0.22\ \mathrm{SE}$, *P* = 0.046; significance evaluated via randomization tests; see Materials and Methods).

When visualised on a polar plot, our behavioral metrics showed bimodal distributions in their nonlinear patterns (Fig. 3*C*). This motivated us to more carefully characterize this cross-participant heterogeneity in behavioral patterns in the following section.

### Distinct Patterns in the Impact of Bottom-Up Stimulus Drive on Behavior

Instead of a normal distribution, we observed multiple modes in behavioral patterns in response to bottom-up alpha band stimulus drive (compare the filled histograms that reflect observed data to the unfilled histograms that reflect a unimodal distribution with the same mean in Figure 3*C*; circular Kolmogorov–Smirnov tests against 1000 Von Mises distributions with equal mean and variance: sensitivity: *P* < 0.01 for 1000/1000; bias: *P* < 0.05 for 968/1000; hit rates: *P* < 0.05 for 989/1000; FA: *P* < 0.05 for 974/1000; RTs: *P* < 0.05 for 966/1000; see Materials and Methods). To understand these multimodal behavioral distributions in response to alpha drive, we used an unsupervised k-means clustering algorithm to divide participants into groups (see Materials and Methods). Separation into 2 groups produced the most parsimonious account of the data as assessed through the “elbow method” and silhouette values (Supplementary Fig. 2; 2 vs. 3–6 groups silhouette value *t*(51)‘s = 3.4, 3.1, 2.6, 3.9; *P*’s = 0.001, 0.003, 0.01, 0.0003).

**Figure 4:**
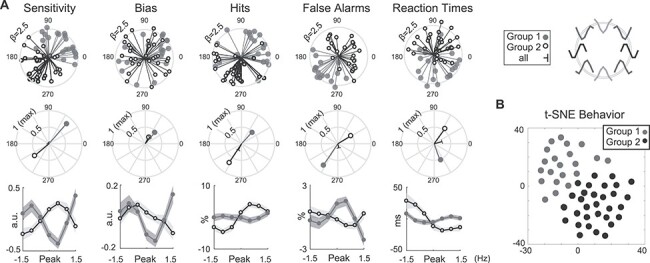
Behavioral groupings. (*A*) Polar plot interpretation guide (left panel) is plotted alongside single participant vectors (top row), where participants are color coded according to their behavioral group (Group 1 is gray; Group 2 is black and white). The outermost ring indicates a combined nonlinear regressor $\square$ vector length of 2.5. Middle row shows group average vectors for Group 1, Group 2, and all participants (black), where the outermost radial ring is the largest possible vector length. Bottom row shows the behavioral patterns separately for each participant group. Errorbars show between-subject SEM. Between-group means are removed for comparison purposes; see Supplementary Figure 1*B* for figures preserving group means. (*B*) *t*-SNE plot for visualizing subject behavior in a 2-dimensional space. Participants were color coded by group membership determined with the *k*-means clustering algorithm.

This participant partitioning resulted in 21 participants in Group 1 (gray circles) and 31 participants in Group 2 (white circles; Fig. 4*A*; see Materials and Methods; all unreported behavioral effects in this and the following paragraph indicate a failure to reach statistical significance). Bottom-up drive at peak alpha frequency reduced sensitivity, largely through increased false alarm rates, for participants in Group 1 (sensitivity: ${\beta}_{\mathrm{cos}}=1.03$, *P* = 0.0008; false alarms ${\beta}_{\mathrm{cos}}=0.59$, *P* = 0.003; Fig. 4*A* bottom row). When receiving bottom-up drive above at compared with below peak alpha, these participants also showed reduced sensitivity as a result of both lower hit rates and more false alarms, as well as slower reaction times (hit rates ${\beta}_{\mathrm{sin}}=0.70$, *P* = 0.028; RT: ${\beta}_{\mathrm{sin}}=-0.93$, *P* = 0.001; false alarms ${\beta}_{\mathrm{sin}}=-1.2$, *P* < 10^−15^; sensitivity: ${\beta}_{\mathrm{sin}}=1.18$, *P* < 10^−15^; Fig. 4*A*). Thus, multiple related behavioral metrics indicate impaired perceptual performance when receiving alpha drive at or above peak alpha for the first group of subjects.

On the other hand, participants in Group 2 showed higher sensitivity, driven by higher hit rates and fewer false alarms, when driven at, versus away from, the endogenous alpha frequency (sensitivity ${\beta}_{\mathrm{cos}}=-0.71$, *P* < 10^−15^; hit rate ${\beta}_{\mathrm{cos}}=-0.65$, *P* = 0.003; false alarms: ${\beta}_{\mathrm{cos}}=0.59$, *P* = 0.003). Additionally, drive above peak alpha frequency increased sensitivity by increasing hit rates and reducing false alarms, as well as resulting in faster RTs (sensitivity: ${\beta}_{\mathrm{sin}}=-0.63$, *P* = 0.0016; hit rate ${\beta}_{\mathrm{sin}}=-0.93$, *P* < 10^−15^; ${\beta}_{\mathrm{lin}}=0.70$, *P* = 0.27; false alarms: ${\beta}_{\mathrm{lin}}=-0.77$, *P* = 0.02; RT: ${\beta}_{\mathrm{sin}}=0.83$, *P* = 0.0004). Thus, several related behavioral metrics suggest that bottom-up drive at or above each participant’s peak alpha frequency generally enhanced performance in the second group of participants (Fig. 4*A*).

We confirmed that these participant groups did not differ on their overall response to bottom-up stimulus drive (RESS response ratios for Group 1 = 4.18 ± 0.25 and Group 2 = 4.32 ± 0.39 SE for; 2-way ANOVA on RESS response ratios with Group and Experiment version as factors: main effect of participant group: *F*(1,51) <10^−4^, *P* = 0.99) or on the experiment type the participant participated in (assessed using both ANOVA and Chi-squared tests: experiment-by-participant group interaction in 2-way repeated measures ANOVA: *F*(1,51) = 0.58, *P* = 0.45; *χ*^2^(1,51) = 0.008, *P* = 0.78, significance based on 10 000 randomized participant groupings).

### Behavioral Groupings Are Linked to the Frequency of the Endogenous Alpha Rhythm

We next asked whether the source of these divergent patterns in behavioral response to bottom-up drive could be related to either the amplitude or frequency of the endogenous alpha oscillation. Importantly, note that participants were grouped based only on their behavioral response to bottom-up alpha stimulation (Fig. 3).

**Figure 5:**
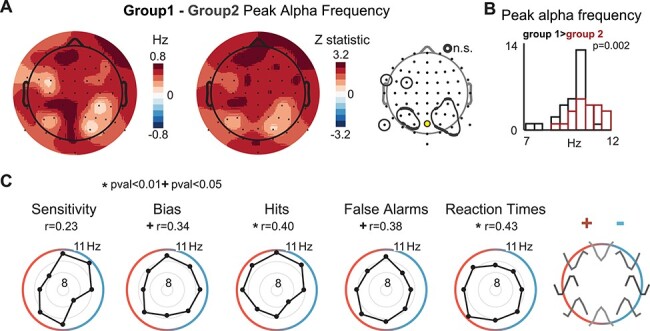
Relationship between behavior and peak alpha frequency (*A*) Behavioral group was associated with peak alpha frequency. Group 1 showed higher peak alpha frequencies over all electrodes (left scalp map). The center scalp map shows *z*-statistics for each electrode, and the right most scalp map circles electrode areas that were not significant after FDR correcting *P* values obtained through comparison with 10 000 randomizations of participant group at 0.05. (*B*) Peak alpha for the 2 groups at channelOz (indicated with yellow circle in topographic map on right side of panel *A*. Group1 is in black; group 2 is outlined in red. (*C*) Circular-linear correlations between single participant nonlinear vectors and their peak alpha frequency were computed for each behavioral metric. Black dots indicate averages over 45-degree bins, gray rings indicate steps of 1 Hz. Rho correlation values significant at *P* < 0.01 are accompanied by ^*^, *P* < 0.05. Guide to the right indicates the observed behavioral pattern, be it an increase (red) or decrease (blue) with stimulation above peak alpha frequency.

We found no difference between the overall amplitude of resting alpha between Group 1 and Group 2 in any electrode (Wilcoxon Rank Sum test significance determined by comparing z-statistics to 10 000 *z*-statistics obtained after randomizing group assignment: *P*’s ≥ 0.28, −0.67 ≤ z’s ≤ 1.15; electrode Oz; see Materials and Methods). However, there was a significant difference in peak alpha frequency between the groups: Participants in Group 1 had a peak frequency of $10.49\pm 0.15\ Hz$ compared with 9.81 ± 0.16 Hz in Group 2 (mean ± SEM; *z* = 2.69, *P* = 0.004; electrode Oz). In addition, participants in Group 1 had numerically higher peak alpha frequencies over all electrodes, with an average of 10.53 Hz compared with 10.02 Hz for Group 2, and this elevation was significant in 52 out of 64 electrodes after FDR correction (Fig. 5*A*; Wilcoxon Rank Sum test significance comparing z-statistics to 10 000 *z*-statistics obtained after randomizing group assignment after FDR correction at 0.05: 0.303–0.764 Hz, 1.8 ≤ z‘s ≤ 2.92, 0.0015 ≤ *P*’s ≤ 0.04; all frequency differences ≥0.1 Hz). We repeated this analysis using different interpolation and regression pipelines and found consistent results (Supplementary Table 1; see Materials and Methods). Finally, we note that since sensitivity and criterion are computed from hit and false alarm rates, several of the behavioral metrics are interrelated. As such, we verified that peak alpha frequency differs with behavioral groupings based solely on the sensitivity metric (Supplementary Fig. 5).

Thus, participants that performed worse when receiving stimulus drive at or above their peak alpha frequency had naturally faster endogenous alpha oscillations (Group 1), while the other group of participants that actually benefited from alpha drive at or above their peak frequency had naturally slower endogenous alpha oscillations (Group 2).

### Nonlinear Patterns in Behavior Are Predicted by Endogenous Alpha Frequency

While these groupings are intriguing, we next investigated whether the behavioral pattern of response to bottom-up stimulus drive depended on alpha frequency in a continuous manner. We did this by computing circular-linear correlations between peak alpha frequency and the angle of each participant’s nonlinear behavioral pattern $\left(\rm{computed \ as } \ {\tan}^{-1} \quad \frac{\beta sin}{\beta cos}\right)$. Peak alpha frequency was indeed continuously associated with patterns of hit rates and RTs in the same manner as previously described—subjects with higher endogenous frequencies displayed lower hit rates and faster RTs with exogenous drive at peak alpha (Fig. 5*C*; hit rate: *r* = 0.404, *P* = 0.01; RT: *r* = 0.425, *P* = 0.006). We also observed weaker correlations for bias and false alarms (bias: *r* = 0.34, *P* = 0.048; false alarms: *r* = 0.382, *P* = 0.038; *P* values based on comparison to a null correlation distribution obtained by randomizing peak alpha frequency 10 000 times). Thus, these correlations are consistent with the finding that participants with fast endogenous alpha oscillations performed worse when driven at or above peak alpha, while participants with slow endogenous oscillations showed the opposite pattern. Interestingly, peak alpha frequency was not correlated with the magnitude of the linear trend term (*P*’s > 0.3 for all behavioral metrics). Thus, we observed an association between nonlinear perceptual patterns in response to alpha drive and peak alpha frequency but did not observe a relationship between alpha frequency and the linear term or between behavior and alpha power (Supplementary Fig. 6). We also replicated these relationships with a polynomial model of behavior (Supplementary Table 2). This indicates that instead of depending on 2 specific groups, there is a continuous relationship between endogenous alpha frequency and the impact of bottom-up stimulus drive on behavior.

### State Space Velocity Is Modulated by Interactions between Endogenous and Driven Frequencies

**Figure 6:**
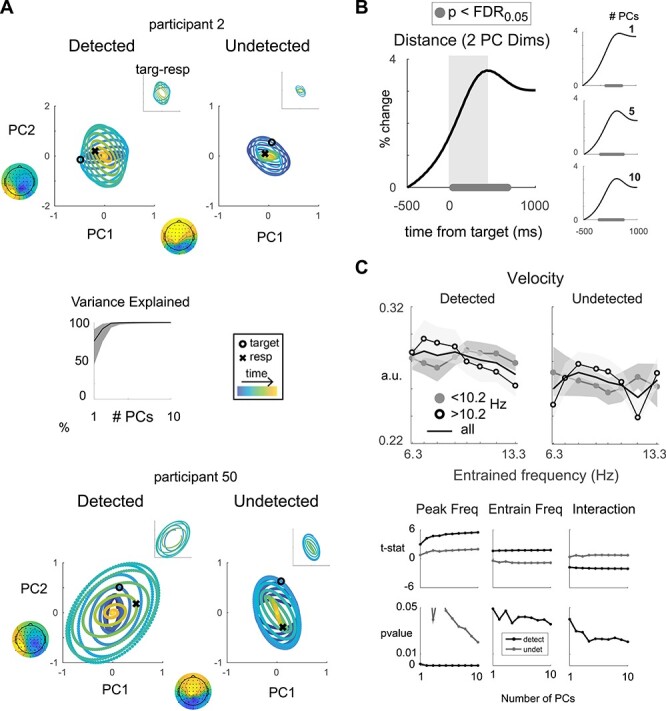
Endogenous alpha state space. (*A*) Top and bottom rows show average trajectories in the PC state space, for example participants on detected (left panels) and undetected (right panels) trials. Black circles denote target onset, black X denotes average reaction time, and timepoints start 500 ms before the target (purple) and end 1000 ms after the target (yellow). Variance explained is plotted for all participants as a function of the number of PCs (middle panel; shaded area indicates minimum to maximum across participants). Topographic maps corresponding to the first and second PCs are included for these 2 participants to display their physiological basis. Insets show the trajectories only from stimulus onset to average time of response. (*B*) Percent change in Euclidean distance traveled on detected—undetected trials plotted from 500 ms before the target to 1000 ms after the target. Black dots indicate timepoints with *t*-statistics that remained significant after comparison with 5000 *t*-statistics computed from randomized condition labels and FDR correction at *P* < 0.01. Left panel: Difference in distance traveled in the 2-dimensional PC state space. Gray shaded rectangle indicates timepoints used for analysis in *C*. Right panel: Difference in distances traveled computed in 1-, 5-, and 10-dimensional state spaces are also plotted, and significance is computed and plotted identically as for left panel. (*C*) Velocity in a 500 ms posttarget epoch on trials in which the target was detected (top left panel) and on trials when it was not (top right panel). Participants were median split based on peak alpha frequency to display the interaction between driven frequency and peak alpha frequency. Errorbars indicated standard error. *T*-values (middle row) from linear mixed effects models fit separately for detected (light gray) and undetected (dark gray) velocities. The *x*-axis displays models fit separately for PCs 1–10. Bottom panel shows *P* values determined from 5000 randomizations of velocity between participants.

So far, we found that distinct behavioral patterns in response to bottom-up stimulus drive are linked to the frequency of each subject’s endogenous alpha rhythm. We next explored the possibility that the observed subject/group distinctions in behavior are related to differential dynamics in the endogenous alpha oscillation under slow versus fast exogenous alpha drive. To test this, we formed a state space from the principle components (PCs) of variation in the independent resting session data (filtered ±1 Hz around each participant’s peak alpha frequency using a third-order zero-phase digital Butterworth filter; see Materials and Methods). Then, we projected the RESS scalp maps out of the task-engaged EEG data to attenuate the influence of the bottom-up drive (see Materials and Methods), and subsequently filtered these data around each participant’s peak alpha frequency (±1 Hz around peak alpha frequency, third-order zero-phase digital Butterworth filter; see Materials and Methods). We then projected the task engaged data into the state space formed from the first 1–10 PCs that explained the most variance in the resting data to ensure any observed effects were robust to the dimensionality of the state space (Fig. 6*A* right panel shows percent variance explained; analysis based on Baria et al. 2017, see Materials and Methods). It is important to note that we chose to form the state space from the independent resting data so that projection of the task-related data into this state space, and any resulting conclusions, would not be biased by dynamics incurred by alpha drive during the task.

We found that trajectories for trials on which the target was detected traversed a greater distance in the state space compared with trials where the target went undetected (% change in Euclidean distance; Fig. 6*A*,*B*; timepoints from 33 to 642 ms after target presentation survive FDR correction at 0.05 using 2 PCs: 2.4 ≤ *t*(51)s ≤ 3.0). We summarized this pattern more compactly by computing state space velocity (e.g. distance per unit time), which we found was faster when the target was detected (500 ms window indicated by gray shaded area in Fig. 6*B*; 2–10 PCs: 3.0 ≤ *t*(51)‘s ≤ 3.8, *P*’s ≤ 0.002; nonsignificant for 1 PC: *t*(51) = 1.3, *P* = 0.25). These results are consistent with previous work positing that the rapid and transient neural dynamics observed with conscious perception may be computationally advantageous because they are both robust to differences in the initial brain state and naturally contextualize incoming stimuli (Maass et al. 2002; Misha et al. 2008; Buonomano and Maass 2009; Baria et al. 2017).

Given our finding that perception was impaired when participants with fast endogenous alpha oscillations were driven at or above their peak frequency, we next tested whether high-frequency alpha drive resulted in decreased posttarget state space velocity for participants with high peak frequencies (and vice versa for low-frequency participants; velocity averaged over 500 ms as indicated by gray shading in Fig. 6*B*). Indeed, we observed a significant interaction between endogenous and driven frequency. As observed before, low-frequency alpha drive resulted in more rapid state space traversal for participants with fast endogenous oscillations, the same low-frequency drive resulted in slower state space traversal for participants with naturally slow endogenous oscillations (Fig. 6*C* top left panel). This interaction between endogenous and exogenously driven frequency was observed on trials where the target was detected, but not on trials where the target was undetected (Fig. 6*C* bottom right panels; linear mixed effects model predicting velocity computed using 1–10 PCs; detected interaction: −2.1 ≤ *t*(412)s ≤ −1.8, *P*’s ≤ 0.04; undetected interaction: 0.37 < *t*(412)s < 0.73, *P*’s ≥ 0.2; ME of drive—detected: 1.6 ≤ *t*(412)s ≤ 1.8, *P*’s ≤ 0.049; undetected: *P*’s ≥ 0.18; see Materials and Methods). We note this interaction was not present in alpha band phase locking or the amplitude of the ERP, indicating a selective impact of alpha drive on endogenous state space dynamics (interaction effect for detected/undetected trials: phase locking index: *t*(412)‘s = −1.3/−0.02, *P*’s = 0.18/0.98; ERP: *t*(412)s = −0.4/−0.07, *P*’s = 0.71/0.95; see Materials and Methods). Additionally, state space velocity increased with peak frequency on all trials, consistent with previous results showing enhanced behavioral performance for subjects with faster alpha oscillations (Fig. 6*C*: ME of peak frequency—detected: 3 ≤ *t*(412)s ≤ 5.5, *P*’s ≤ 0.001 for PCs 1–10; undetected: 1.7 ≤ *t*(412)s ≤ 2, *P*’s ≤ 0.048 for 3, 5–10 PCs) (Klimesch et al. 1993; Angelakis et al. 2004; Richard Clark et al. 2004; Cecere et al. 2015). Again, this impact of peak frequency was not observed in phase locking or ERP amplitude (PLI: *P*’s ≥ 0.11, ERP: *P*’s ≥ 0.77). Thus, high-frequency alpha band drive in subjects with fast endogenous alpha oscillations leads to slower state space traversal and worse performance, while the same high-frequency drive leads to more efficient state space traversal in subjects with naturally slow alpha oscillations.

## Discussion

Here, we used a flickering stimulus to drive bottom-up rhythms at multiple frequencies in the alpha band while participants performed a visual detection task. This manipulation successfully drove posterior rhythms that could be isolated from the endogenous alpha oscillation via source separation, allowing us to investigate the combined effects of stimulus-driven rhythms and endogenous alpha oscillations on perception.

When perceptual performance was aligned to each participant’s endogenous alpha frequency, we observed different perceptual patterns in response to bottom-up drive that were systematically associated with the frequency of each participant’s endogenous alpha rhythm. Specifically, participants with faster endogenous alpha oscillations showed impaired behavior when driven at or above this endogenous frequency, while participants with slower alpha oscillations showed enhanced behavior when driven at or above their endogenous alpha frequency. Thus, the perceptual impact of bottom-up alpha drive depends on individual endogenous alpha frequency.

We next investigated whether these distinct behavioral responses could be explained via changes in the dynamics of the endogenous alpha oscillation under different frequencies of exogenous drive. First, we found an association between rapid changes in the state of the endogenous alpha oscillation and more accurate perceptual processing, consistent with previous results (Baria et al. 2017). Using this metric, we also found that naturally fast endogenous alpha oscillations traversed the state space slower under high-frequency bottom-up drive, which may reflect a reduction in efficiency based on its association to impaired behavioral performance. This same high-frequency drive also may have led to more efficient state space traversal in participants with slow alpha rhythms, as inferred from improved behavioral performance (and see Baria et al. 2017). This interaction between endogenous and driven alpha frequency suggests that driving neural circuits at alpha band frequencies away from the peak endogenous frequency leads to more efficient oscillatory dynamics. Further work with spatially targeted neural recordings, such as local field potentials, will be necessary to characterize the spatial extent of these exogenously driven rhythms and the circuit-level mechanisms of their interaction with generators of the endogenous alpha oscillation.

Overall, we found a symmetric relationship between the frequency of endogenous alpha oscillations and exogenous stimulus drive. Low-frequency alpha drive leads to enhanced perception and more efficient dynamics when endogenous alpha oscillations are naturally fast. In contrast, high-frequency alpha drive improves perception—potentially via more efficient dynamics—when endogenous oscillations are slow (Figs 4–6). Interestingly, this suggests that driving early visual circuits *away* from the endogenous alpha frequency while remaining within the frequency bounds of the alpha band may be beneficial, perhaps by decoupling the bottom-up circuits that process incoming stimuli from the endogenous alpha oscillation. Alternatively, this central tendency may suggest that the middle of the alpha band is an optimal dynamic range for bottom-up stimulus processing and visual perception. We speculate that this convergence could be due to the stereotypy of the large-scale anatomical wiring of the visual system (Van Essen and Maunsell 1983; Felleman and Van Essen 1991; Van Essen 2005; Takemura et al. 2016) and the role of network size in the temporal dynamics of information propagation (Draguhn et al. 2004; Buzsáki 2006; Lea-Carnall et al. 2016).

Further work is needed to address the intriguing possibility that a similar central tendency in oscillatory frequency governs the dynamical mechanisms by which endogenous alpha oscillations support visual perception. For example, most prior work concerning endogenous alpha oscillations focuses on their role in inhibitory processing (For review, see Klimesch et al. 2007; Palva and Palva 2007; Jensen and Mazaheri 2010; Başar and Güntekin 2012; Jensen et al. 2012; Zauner et al. 2012). However, it is unknown if endogenous frequency factors into the dominant alpha-as-inhibition theoretical framework, as few of the empirical reports forming the basis of the inhibition hypothesis consider peak alpha frequency as a key variable (Nelli et al. 2017). We speculate that considering alpha frequency could help unify the inhibitory framework with seemingly disparate results that also indicate a role for alpha oscillations in enhanced processing (for review, see Foster and Awh 2019). Likewise, studies using casual manipulations of brain rhythms, such as SSVEPs, rhythmic microstimulation, or optogenetics, should more carefully consider how exogenous manipulations interact with endogenous oscillations. A failure to do so may result in effects that are idiosyncratically tied to the exact choice of stimulation frequency with respect to endogenous oscillatory activity.

## Funding

NDSEG graduate fellowship (to S.N.); James S. McDonnell Foundation (NIH R01-EY025872 and a Scholar Award to J.T.S).

## Notes

*Conflict of Interest:* The authors declare no competing financial interests or conflicts of interest.

## Supplementary Material

Supplementary_Materials_Final_2_tgab032
