# Supplementary material for: Individual Alpha Frequency Determines the Impact of Bottom-Up Drive on Visual Processing
Source: Cereb Cortex Commun. 2021 Apr 26;2(2):tgab032. doi: 10.1093/texcom/tgab032 (PMC8171796; doi:10.1093/texcom/tgab032)
Supplement: Supplementary_Materials_Final_2_tgab032 [file supplementary_materials_final_2_tgab032.pdf]

Supplementary Materials

Supplemental Figures

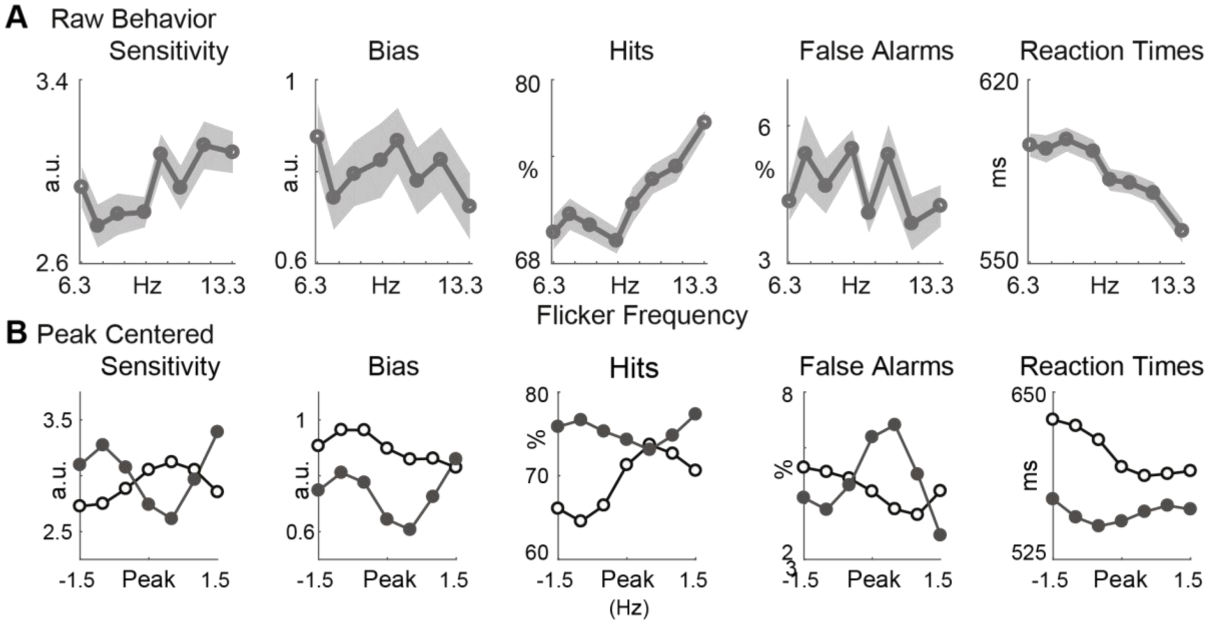

Figure S1: A) Behavioral metrics as a function of flicker frequency averaged across all participants. Dot's indicate mean values over all participants, shaded areas indicate between subjects SEM. **B)** The peak centered data preserving between-group differences for comparison with Figure 4.

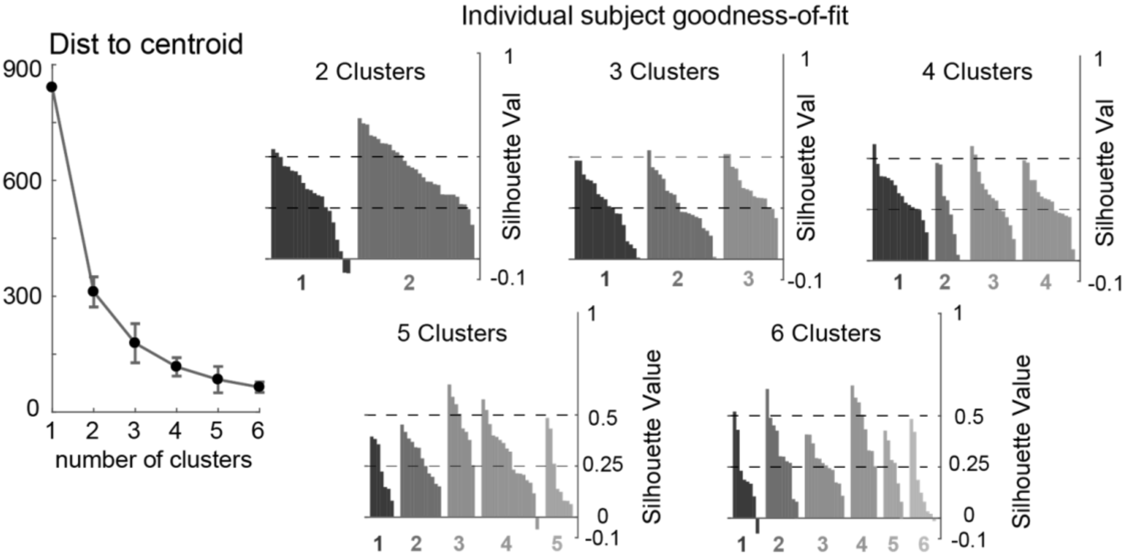

Figure S2: K-means cluster number determination. Left Panel: The mean distance of each point to the center of the cluster for different cluster numbers. Dots indicate mean, error bars indicate standard deviation over clusters (hence no bar for 1 cluster). Right Panel: Individual participant silhouette values were investigated as a goodness of fit measure. We plot from -0.1 to 1 on the x axis and indicate 0.25 and 0.5 with dashed lines. Each bar indicates the silhouette value for one participant and are colored differently according to cluster membership.

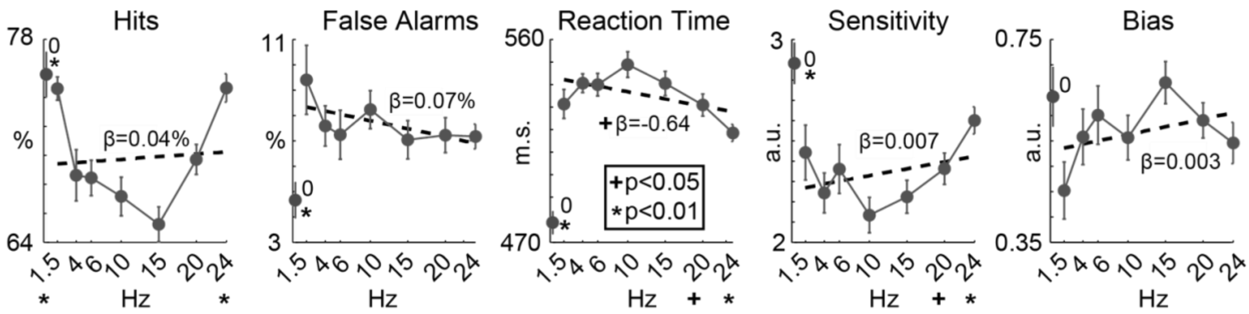

Figure S3: Behavioral results from an experiment that used one driving frequency in the alpha band (10Hz) and seven lower and higher frequencies, along with no stimulation. Specificity of the behavioral effects reported in the main paper to the alpha band indicated by lack of linear behavioral trends over a larger frequency range. These data suggest that the behavioral effects reported in the main text are not simply monotonic changes in behavioral outcomes due to increasing flicker rates. Mean hit rates, false alarm rates, reaction times, sensitivity and bias are plotted, with error bars indicating SEM. Unconnected dots indicate the no stimulation condition. βs and significance indicators from linear regressions are reported next to dotted regression lines. Significance for paired t-tests comparing behavior at 10 Hz and each other frequency are indicated next to the numerical label for each relevant frequency, + indicate p<0.05, * indicate p <0.01.

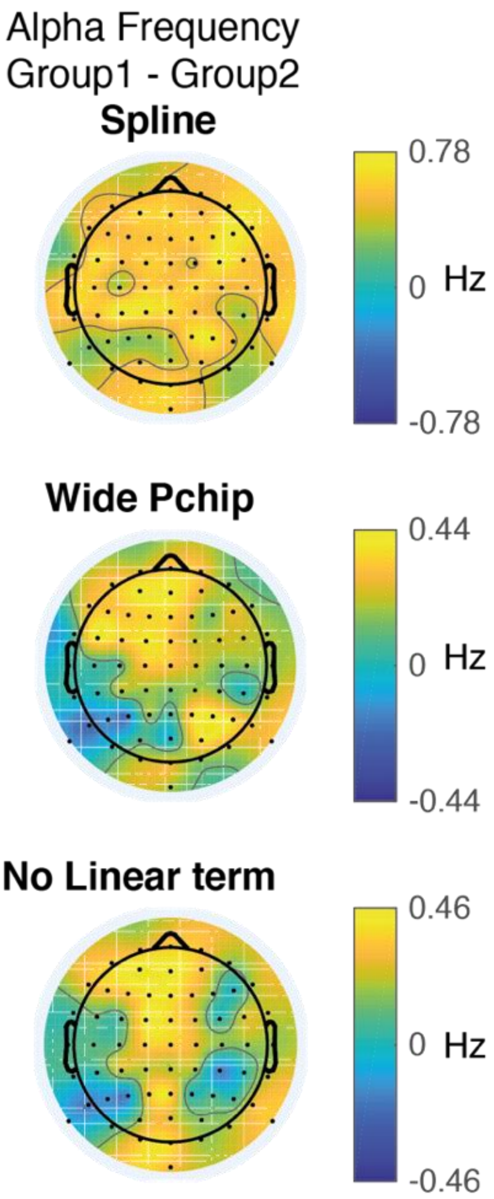

Figure S4: We ran 3 further variants of our entire modelling pipeline to ensure that our results were robust. We peak centered behavioral data, clustered using the k-means algorithm, and then subtracted peak alpha frequencies at all electrodes for the two groups. We plotted the difference in peak alpha frequencies on topographic maps here. These topographic maps demonstrate that our central result that the two groupings in behavioral data correspond to differences in peak alpha frequency are robust to the particular choice of analysis pipeline. In particular, the top plot is showing results obtained using spline instead of p-chip behavioral interpolation for the alpha frequency peak centering. The middle plot is showing results using a p-chip interpolation over a wider range of alpha frequencies. The bottom plot is showing results obtained if behavior is modeled without a linear term, thus reducing the degrees of freedom in the model. We include statistics for these model variants in supplementary table S1.

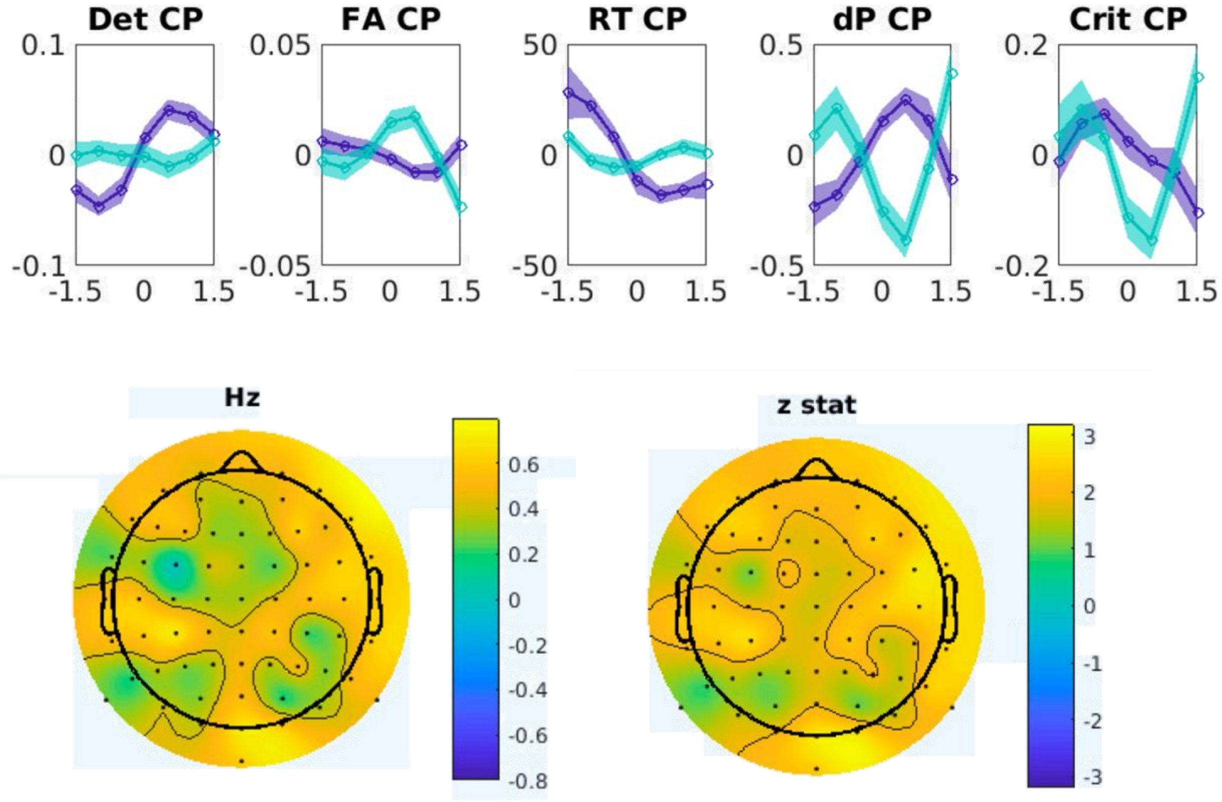

Figure S5: We replicated the result that behavioral groupings are associated with different peak alpha frequencies using just sensitivity as input to the clustering algorithm instead of all 5 behavioral metrics. When the clustering analysis is done just on sensitivity, 49 of the 52 subjects are placed into the same cluster. Additionally, the topoplots of the difference in peak alpha between the two groups looks similar to Figure 5, with mean values of 10.46 ± 0.77 Hz and 9.76 ± 0.92 Hz at electrode OZ (compared with figure 5 and the stats found on page 26-27: 10.49 ± 0.15Hz and 9.81 ± 0.16 Hz).

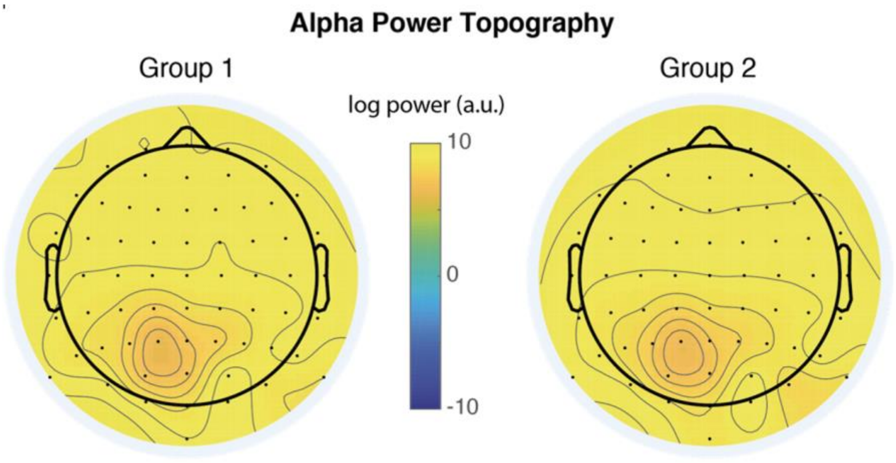

Figure S6: A ranksum test at each electrode did not reveal any electrodes at which the amplitude was significantly different between the two groups at p<0.05.

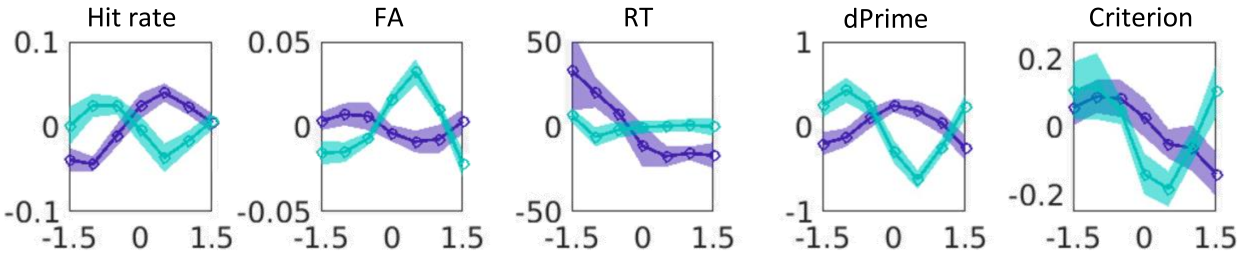

Figure S7 We noted a considerable amount of variance in behavior, and so excluded participants with hit rate below 65% and above 85% (roughly, ± 10% from the target detection rate of 75%). Doing this resulted in the removal of 27 participants (52% of our sample). We still observed similar behavioral patterns across the two groups as plotted above (compare with figure 4, page 26).

Peak centring Scheme	Behavioural regressors	Group 1 (Hz)	Group 2 (Hz)	G1 – G2
Original Pchip<br>±1.5 Hz	Cosine, Sine, Linear, Constant	10.53	10.02	t(63) = 27.2<br>$p < 10^{-14}$
Spline<br>±1.5 Hz	Cosine, Sine, Linear, Constant	10.54	10.03	t(63) = 32.2<br>$p < 10^{-14}$
Wide Pchip<br>±2 Hz	Cosine, Sine, Linear, Constant	10.33	10.13	t(63) = 9.9<br>$p < 10^{-13}$
No linear Pchip<br>±1.5 Hz	Cosine, Sine, Constant	10.33	10.14	t(63) = 9.6<br>$p < 10^{-13}$

Table S1: Impact of analysis pipeline on frequency averaged over the scalp for each participant group. In addition to the originally reported p-chip interpolation algorithm from ±1.5 Hz and four regressors, we included 3 more analysis pipelines (First Column). In this first variant of our analysis pipeline, we used spline interpolation (which considers all data points) and did not change the interpolation range or the regressors of interest (Second Column). The unsupervised k-means clustering algorithm partitioned 49 of the 52 participants into the same group as our original analysis, and we group 1 had an elevated peak alpha frequency compared with group 2 (averaged over entire scalp, last three columns). This indicates that the exact choice of interpolation algorithm does not alter our conclusions, and that an algorithm considering all data points yields similar results. We ran a version using the pchip algorithm to achieve peak alpha centering, as in the manuscript, but with a wider interpolation window (-2 to +2 Hz instead of -1.5 to +1.5 Hz). We again saw increased alpha frequency in group 1 compared to group 2, and the unsupervised algorithm (k-means clustering) partitioned 45 of the 52 participants into the same groups on the basis of their behavioral data. Finally, we ran a version in which we did not include the linear term and 47 of the 52 participants were partitioned into the same groups as reported in the original pipeline, and we again observed increased alpha frequency in group 1 compared to group 2.

Pearson Correlations with Peak Alpha	Sensitivity	Crit	Hits	FA	RT
3rd	n.s.	-0.30 +	0.38*	n.s.	n.s.
2nd	n.s.	-0.35 +	n.s.	0.27+	n.s.
Linear	n.s.	n.s.	-0.37 *	n.s.	n.s.
Constant	n.s.	0.35 +	n.s.	-0.27 +	n.s.
Cos	n.s.	0.31 +	n.s.	-0.31 +	-0.33 +
Sin	n.s.	n.s.	0.38 *	n.s.	-0.29 +
Linear	n.s.	n.s.	n.s.	n.s.	n.s.
Constant	n.s.	n.s.	n.s.	n.s.	0.41 *

Table S2: Linear correlations between model regression parameters and behavioral metrics. The circular correlations reported in the text give a wholistic view of how peak alpha and behavior are correlated, while here they are broken down for detailed comparison. In white are the correlations between the terms of a third order polynomial model fit to behavior and peak alpha frequency, in gray are correlations using terms from the full sinusoidal model reported in the text. + indicates $p < 0.05$, * indicates $p < 0.01$. We note that fit errors were lower in the sinusoidal model than the polynomial approach ($t(33)$s: Sensitivity = -3.69, RT = -2.99, Hits = -5.95, Criterion = -4.17, FA=-2.91).

Supplementary Results

Experimental-Wise Differences: Continuous vs trial-by-trial

We observed significant responses to alpha-band drive in both experiment versions, although, as may be expected, amplitude was higher in the continuous version, with SNR = 5.27 ±0.3 SE vs 2.7 ± 0.03 SE in the trial-wise version (Two-way ANOVA with experiment and k-means participant-group as factors, $F(1,51) = 104.48$, $p<10^{-12}$).

Given this correlation between experiment type and because of the different structure of the two behavioral tasks that were used, we evaluated participants in each experiment for differences before combining the data in the main analyses (Pearson correlation = -0.83, $p < 10^{-13}$). Importantly, although experiment type was correlated with SSVEP response, behavioral performance and experiment type was not predictive of SSVEP entrainment (2-way ANOVA interactions $F(1,51)<0.35$, p's>0.55 for all behavioral metrics). Given this lack of interaction and significant response to drive in both experiments, we collapsed all data across experiment subtype (paired t-tests of RESS SNR against 1: Continuous 7.9 ≤ t(30)'s ≤13.7, p's<10^{-8}; Trial-wise 4.3 ≤ t(20)'s ≤ 6.1, p's<0.001).

We also assessed whether experiment type was associated with behavioral participant groupings and found that experiment version did not interact with k-means participant grouping (Two-way repeated measures ANOVA: Experiment X Participant-group interaction $F(1,51) = 0.58$, $p=0.45$).
